# Prognostic value of testosterone for the castration-resistant prostate cancer patients: a systematic review and meta-analysis

**DOI:** 10.1007/s10147-020-01747-1

**Published:** 2020-07-17

**Authors:** Noriyoshi Miura, Keiichiro Mori, Hadi Mostafaei, Fahad Quhal, Reza Sari Motlagh, Mohammad Abufaraj, Benjamin Pradere, Abdulmajeed Aydh, Ekaterina Laukhtina, David D’Andrea, Takashi Saika, Shahrokh F. Shariat

**Affiliations:** 1 Department of Urology, Comprehensive Cancer Center, Medical University of Vienna, Vienna, Austria; 2 Department of Urology, Ehime University Graduate School of Medicine, Ehime, Japan; 3 Department of Urology, The Jikei University School of Medicine, Tokyo, Japan; 4 Research Center for Evidence based Medicine, Tabriz University of Medical Sciences, Tabriz, Iran; 5 Department of Urology, King Fahad Specialist Hospital, Dammam, Saudi Arabia; 6 Division of Urology, Department of Special Surgery, Jordan University Hospital, The University of Jordan, Amman, Jordan; 7 Department of Urology, University Hospital of Tours, Tours, France; 8 King Faisal Medical City, Abha, Saudi Arabia; 9 Institute for Urology and Reproductive Health, Sechenov University, Moscow, Russia; 10 Department of Urology, Weill Cornell Medical College, New York, NY USA; 11 Department of Urology, University of Texas Southwestern, Dallas, Texas USA; 12 Karl Landsteiner Institute of Urology and Andrology, Währinger Gürtel 18-20, 1090 Vienna, Austria; 13 Department of Urology, Second Faculty of Medicine, Charles University, Prague, Czech Republic; 14 European Association of Urology Research Foundation, Arnhem, The Netherlands

**Keywords:** Prostate cancer, Testosterone, Meta-analysis, Androgen receptor-targeted agents, Castration-resistant

## Abstract

**Introduction:**

This systematic review and meta-analysis aimed to assess the prognostic value of testosterone in patients with castration-resistant prostate cancer (CRPC).

**Materials and methods:**

PubMed, Web of Science, and Scopus databases were systematically searched until December 2019, according to the Preferred Reporting Items for Systemic Review and Meta-analysis statement. The endpoints were progression-free survival (PFS) and overall survival (OS).

**Results:**

We identified 11 articles with 4206 patients for systematic review and nine articles with 4136 patients for meta-analysis. Higher testosterone levels were significantly associated with better OS (pooled HR 0.74, 95% CI 0.58–0.95) and better PFS (pooled HR 0.51, 95% CI 0.30–0.87). Subgroup analyses based on the treatment type revealed that higher testosterone levels were significantly associated with better OS in CRPC patients treated with androgen receptor-targeted agents (ARTAs) (pooled HR 0.64, 95% CI 0.55–0.75), but not in those treated with chemotherapy (pooled HR 0.78, 95% CI 0.53–1.14).

**Conclusion:**

This meta-analysis demonstrated that the PFS and OS were significantly greater in patients with CRPC in those with higher testosterone levels than that of those with lower testosterone levels. In the subgroup analyses, lower testosterone levels were a consistently poor prognostic factor for OS in patients treated with ARTAs, but not in those treated with chemotherapy. Therefore, higher testosterone levels could be a useful biomarker to identify patient subgroups in which ARTAs should be preferentially recommended in the CRPC setting.

**Electronic supplementary material:**

The online version of this article (10.1007/s10147-020-01747-1) contains supplementary material, which is available to authorized users.

## Introduction

Testosterone is the main growth stimulator for hormone-sensitive prostate cancer (PC). Androgen-deprivation therapy (ADT) is the mainstay treatment for some advanced and almost all metastatic hormone-sensitive PC [1]. Despite early efficacy, eventually, tumors progress to a castration-resistant state during ADT despite serum testosterone levels below castration levels (< 50 ng/dL) [1]. Recently, evidence regarding the continuous control role of androgens and androgen receptor (AR) in this castration-resistant state has been increasing. Pathophysiological mechanisms that may contribute to progression to CRPC are AR gene amplification, AR mutations, increased activity of transcriptional coactivator proteins, stimulation of kinases that directly or indirectly enhance AR responses to low androgen levels, and increased intra-tumoral androgen synthesis [2]. Based on the understanding of these factors in the CRPC pathogenesis, new hormonal axis-targeting androgen receptor-targeted agents (ARTAs) therapies (i.e., enzalutamide and abiraterone acetate) [3, 4] have been developed, tooled, and established as standard therapies for CRPC patients as well as in earlier disease states [1]. Other novel effective therapeutic strategies for CRPC include chemotherapy (docetaxel, cabazitaxel [5], and radium-223 dichloride [6]). One of the challenges has been to understand the optimal sequence of these therapeutic modalities for each individual patient. Testosterone seems to be importance even in CRPC as the level of testosterone may have a higher significance than previously thought. Indeed, the testosterone level to be classified as castrate varies between guidelines and experts [7]. The relationship between serum testosterone and prognosis of PC has been of interest among physicians in recent years [8–10].

Several works of literature to assess the relationship between serum testosterone and PC have been reported. Klap et al reported a comprehensive review of these relationships. The authors concluded that the relationships among serum testosterone levels, the incidence of PC, aggressiveness, and oncological outcomes showed conflicting results and has been controversial [11]. In 2018, Claps et al. reported a systematic review and meta-analysis of the relationship between testosterone levels and PC prognosis based on the articles until September 2017. Focusing on patients with CRPC, serum testosterone levels were found to be significantly associated with reduced risk of progression, although not death [12]. Additionally, the authors included as a limitation the great heterogeneity in the systematic treatments of the patients with CRPC. Moreover, only one of the included studies had assessed the relationship between serum testosterone levels and the overall survival (OS) of patients with CRPC treated with new ARTAs. Therefore, the association between pretreatment serum testosterone levels and prognosis of patients with CRPC treated with new ARTAs and taxane chemotherapies is still unclear.

Several biomarkers have been reported to predict the antitumor response of the new ARTAs and taxane chemotherapy. For example, the presence of visceral metastases, symptomatic disease, and the short response of primary ADT are prognostic factors that favor chemotherapy [13]. However, no definitive guideline to help the choice between the two drugs has been established. Therefore, we performed a systematic review and meta-analysis to summarize the recent data of the relationship and to assess the prognostic effect of testosterone in patients with CRPC.

## Materials and methods

### Search strategy

The current systematic review and meta-analysis were performed in accordance with the Preferred Reporting Items for Systematic Reviews and Meta-analyses guidelines [14]. We searched PubMed, Web of Science, and Scopus to investigate the prognostic value of testosterone in patients with CRPC until December 2019. Articles published in the English language were only considered. There was no restriction regarding the publication period. The study protocol is registered in the International Prospective Register of Systematic Reviews database (PROSPERO CRD 42020161307).

After an initial screening based on study titles and abstracts, all papers were assessed based on full texts and excluded with reasons when inappropriate; a further evaluation of the appropriateness of the papers based on a full-text revision was performed after data extraction. The following keywords were used in our search strategy: (castration-resistant prostate cancer OR crpc OR hormone refractory prostate cancer OR hrpc) AND (testosterone) AND (survival OR mortality OR progression OR overall survival OR OS OR cancer-specific survival OR CSS OR metastasis-free survival OR MFS). The endpoints of interest were progression-free survival (PFS), cancer-specific survival (CSS), and OS.

The initial screening was performed independently by two investigators based on the titles and abstracts for ineligible reports. The reasons for exclusions were recorded. A full-paper review was performed for potentially relevant reports, and the relevance of the reports was confirmed after the data extraction process. In case of multiple reports of the same cohort, the ones with the most complete data aggregated with the longest follow-up duration were selected. Discrepancies were resolved by consensus or recourse to the senior author.

### Inclusion and exclusion criteria

Studies were included if they included a comparison of patients with CRPC (Patients) treated with any drugs for CRPC with lower testosterone levels (Intervention) and those with higher testosterone levels (Comparison) to assess the prognostic effect of testosterone on PFS, CSS, and OS (Outcome) using multivariable Cox regression analysis (Study design) in nonrandomized, observational, or cohort studies. CRPC was defined as disease progression despite castrate levels of serum testosterone (≤ 50 ng/dL) [1]. PFS was defined as the time interval between the start of treatment and the time of radiological, clinical, or biochemical progression or last tumor evaluation. OS was defined as the time between the first day of treatment and the date of death from any cause or the date of the last follow-up. We excluded articles not in English, reviews, editorials, letters, or case reports.

### Data extraction

The information was independently extracted by two investigators including authors’ names, year of publication, study design, treatment, time of testosterone assessment, testosterone, testosterone cut-off value, number, follow-up duration. Subsequently, the hazard ratios (HRs) and 95% confidence intervals (CIs) of the testosterone associated with each of the outcomes were retrieved. We used the following conversion formula to convert “nmol/L” to “ng/dL” (Testosterone: 0.0347 nmol/L = 1 ng/dL) [15]. HRs were extracted from the multivariable analyses for meta-analysis. Discrepancies were resolved by consensus and/or recourse to the senior author.

### Quality assessment

We assessed the quality of the included studies using the Newcastle–Ottawa Scale (NOS) according to the Cochrane Handbook for systematic reviews of interventions for the included nonrandomised studies [16, 17]. In this meta-analysis, the article quality of cohort studies was assessed as low quality (0–3 points), moderate quality (4–6 points), and high quality (7–9 points). The main confounding factors were identified as the important prognostic factors of PFS and OS. The articles were reviewed to determine the presence of confounders. Studies with scores greater than six were identified as “high quality.”

### Statistical analyses

Forest plots were used to assess multivariate HRs and to obtain summary HRs to elucidate the relationship between higher testosterone levels and PFS and OS. The studies which used only Kaplan–Meier log-rank analysis, univariate Cox proportional hazard regression analysis, or general logistic regression analysis were excluded for meta-analysis. We calculated the corresponding 95% CIs in studies with only HRs and *p* value [18, 19].

Additionally, we performed subgroup analyses for each treatment type, such as ARTAs and chemotherapy. Heterogeneity among the outcomes of the included studies in this meta-analysis was evaluated using the Cochrane’s *Q* test and the *I*^2^ statistics. Significant heterogeneity was indicated by *p* < 0.05 in the Cochran’s *Q* tests and a ratio >50% in *I*^2^ statistics. We used fixed effects models for the calculation of pooled HRs for non-heterogeneity results [20–22]. Publication bias was assessed using funnel plots. All statistical analyses were performed using Stata/MP 14.2 (Stata Corp., College Station, TX); statistical significance level was set at *p* < 0.05.

## Results

### Literature search

**Fig. 1:**
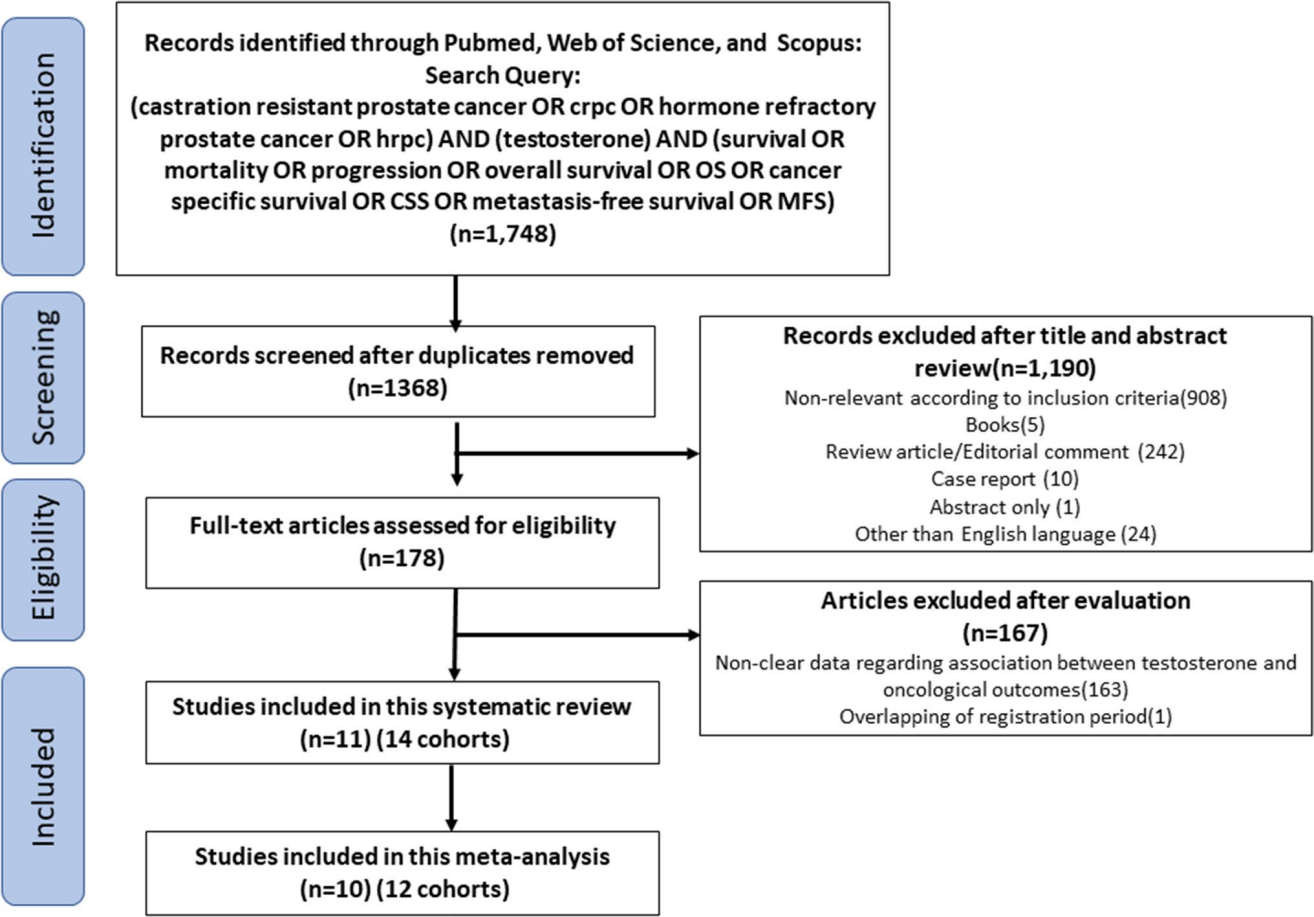
Preferred reporting items for systematic reviews and meta-analyses flow chart for article selection process to analyze the prognostic impact of serum testosterone levels in castration-resistant prostate cancer. The studies in which HR was extracted from the multivariable analysis were included for qualitative meta-analysis

Overall, 1748 publications were identified in the initial search (PubMed, 370; Scopus, 940; Web of Science, 438). Of these, 1570 articles were excluded after screening for duplicates, non-relevant articles according to inclusion criteria, books, reviews, editorial comment, case reports, abstracts without an article, and non-English articles. A full-text review was performed for 178 potentially relevant articles. After evaluating the selection criteria, we identified 11 articles with 4206 patients for systematic review and ten articles with 4136 patients for meta-analysis [8–10, 23–30]. Figure 1 depicts the selection process and list.

### Study characteristics

In the 11 included studies, 14 cohorts were evaluated for endpoints of interest. The extracted data from the 11 studies are outlined in Table 1. Various cut-off values were reported for testosterone levels, with a range of 2.6–14.5 ng/dL and 2.6–13.0 ng/dL for OS and PFS, respectively. Of these included cohorts, those treated with ARTAs were three for OS and seven for PFS. Those treated with chemotherapy were two for OS and one for PFS.

**Table 1:** Study characteristics of 11 studies

Author/year of publication	Study design	Treatment line	Treatment	Timing of T assessment	T (ng/dl) median	Cut-off T values	Daily timing	N	F/U median	HR (95% CI)	Univaraiable/multivaraible
Amstrong (2009) [23]	R	1st	TAX327	Baseline	14.5	Median	NA	1001	20.8	OS: 0.9(0.76–1.08)	M
Bertaglia (2017) [24]	P	2nd	Abi	Post-DTX	14.0	Median	NA	49	23.8	PFS: 0.79(0.43–1.42)	U
de Liano (2014) [25]	R	1st	Chemotherapy	Baseline	11.5	Median	NA	101	20.6	OS: 0.4 (0.6–1)	M
Halabi (2007) [26]	R	1st	Systematic therapies not specified	Baseline	18.0	Continuous variable	NA	1226	33.8	OS: 1.01 (1–1.02) PFS: 1 (0.99–1.01)	M
Hashimoto (2019) [8]	R	Mix	Enz/Abi	Before ARTAs (prior DTX 31 pts)	< 5.0	5 ng/dl	Morning	115	25.7	PFS: 0.3 (0.15–1.61)	M
Hashimoto (2011) [27]	R	1st	Bicalutamide/flutamide	Baseline	10.0	5 ng/dl	NA	30	52.5	PFS: 0.17 (0.05–0.55)	M
Lolli (2019) [28]	R	2nd	Abi	Post-DTX	<2.6	2.6 ng/dl	NA	54	35	OS: 0.46 (0.23–0.92) PFS: 0.49 (0.26–0.94)	M
		2nd	Enz					74		OS: 0.47 (0.2–1.07) PFS: 0.38 (0.18–0.82)	M
Montgomery (2015) [29]	R	2nd	COU-AA 301	Baseline	NR	5 ng/dl	NA	1195	20.2	OS: 0.66 (0.56–0.78)	M
Lin (2012) [30]	R	1st	Ketoconazole	Baseline	14.0	10 ng/dl	NA	160	51.9	PFS: 0.28 (0.13–0.56)	M
Sakamoto (2019) [9]		Mix	Enz/Abi	Baseline	13.0	Medain	NA	107	68.3	OS: 0.91 (0.3–2.64) PFS: 0.31 (0.1–0.93)	OS:U, PFS:M
Shiota (2018) [10]	R	Mix	ENZ	Baseline	3.0	5 ng/dl	AM 8–10	35	NR	PFS: 0.72 (0.19–2.13)	M
		Mix	Abi					21		PFS: 2.50 (0.72–8.69)	U
		Mix	DTX					38		PFS: 2.94 (1.18–7.7)	M

*T* testosterone, *N* number, *F/U* follow-up, *HR* hazard ratio, *CI* confidence interval, *R* retrospective, *P* prospective, *Abi* abiraterone, *Enz* enzalutamide, *DTX* docetaxel, *ARTAs* androgen receptor-targeted agents, *NR* not reported, *OS* overall survival, *PFS* progression-free survival, *M* multivariable, *U* univariable

### Study quality

According to the NOS, ten studies were considered high-quality and one as medium-quality (Supplementary Table 1).

### Meta-analysis

#### Association of testosterone with OS in CRPC

**Fig. 2:**
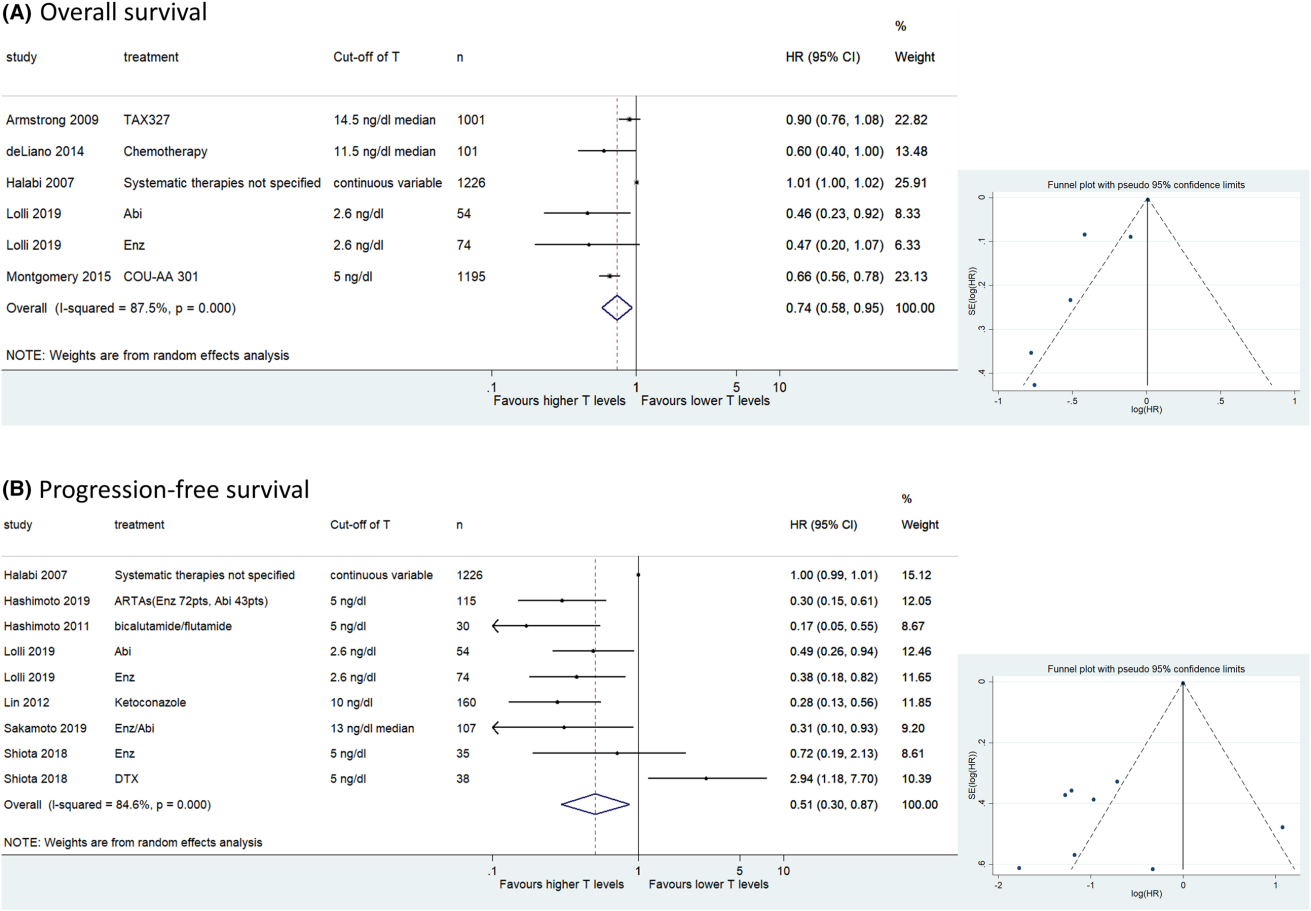
Forest and funnel plots showing the association of serum testosterone levels with oncological outcomes in castration-resistant prostate cancer; **a** overall survival, **b** progression-free survival

We assessed the association between testosterone and OS in six cohorts of retrospective studies including 3641 patients with CRPC. The forest plot (Fig. 2a) revealed that higher testosterone levels according to the cut-off used in each study were significantly associated with better OS (pooled HR 0.74, 95% CI 0.58–0.95; *z* = 2.41; *p* = 0.016). The Cochrane’s *Q* test (*χ*^2^ = 39.87; *p* = 0.000) and *I*^2^ test (*I*^2^ = 87.5%) revealed significant heterogeneity. The funnel plot identified three cohorts over the pseudo-95% CI (Fig. 2a).

#### Association of testosterone with PFS in CRPC

Nine cohorts, including 1839 patients, retrospectively evaluated the association of testosterone level with PFS in CRPC patients. The forest plot (Fig. 2b) revealed that higher testosterone levels were significantly associated with better PFS (pooled HR 0.51, 95% CI 0.30–0.87; *z* = 2.45; *p* = 0.014). The Cochrane’s Q test (χ^2^ = 51.94; *p* = 0.000) and *I*^2^ test (*I*^2^ = 84.6%) revealed significant heterogeneity. The funnel plot identified seven cohorts over the pseudo-95% CI (Fig. 2b).

#### Association of testosterone with OS and PFS in patients with CRPC treated with ARTAs

**Fig. 3:**
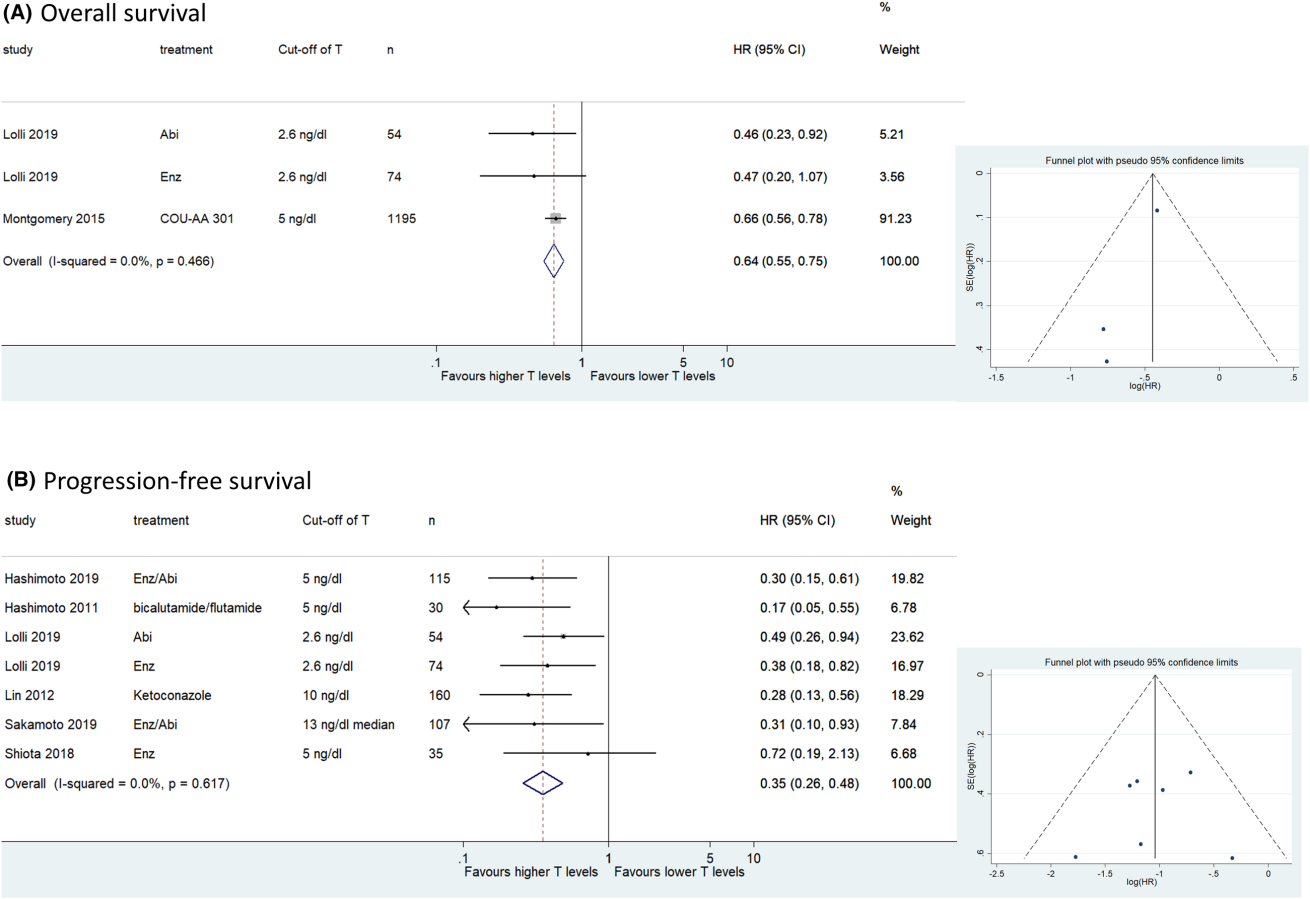
Forest and funnel plots showing the association of serum testosterone levels with oncological outcomes in patients with castration-resistant prostate cancer treated with androgen receptor-targeted agents; **a** overall survival, **b** progression-free survival

Three cohorts including 1323 patients provided data on the association of testosterone with OS in patients with CRPC treated with ARTAs. The forest plot (Fig. 3a) revealed that higher testosterone levels were significantly associated with better OS in patients with CRPC treated with ARTAs (pooled HR 0.64, 95% CI 0.55–0.75; *z* = 5.53; *p* = 0.000). The Cochrane’s *Q* test (*χ*^2^ = 1.53; *p* = 0.466) and *I*^2^ test (*I*^2^ = 0.0%) revealed no significant heterogeneity. The funnel plot identified no cohort over the pseudo-95% CI (Fig. 3a).

Seven cohorts including 575 patients provided data on the association of testosterone with PFS in patients with CRPC treated with ARTAs. The forest plot (Fig. 3b) revealed that the higher testosterone levels were significantly associated with better PFS in patients with CRPC treated with ARTAs (pooled HR 0.35, 95% CI 0.26–0.48; *z* = 6.52; *p* = 0.000). The Cochrane’s *Q* test (*χ*^2^ = 4.45; *p* = 0.617) and *I*^2^ test (*I*^2^ = 0.0%) revealed no significant heterogeneity. No cohort over the pseudo-95% CI (Fig. 3b) was identified by the funnel plot.

#### Association of testosterone with OS and PFS in patients with CRPC treated with chemotherapy

**Fig. 4:**
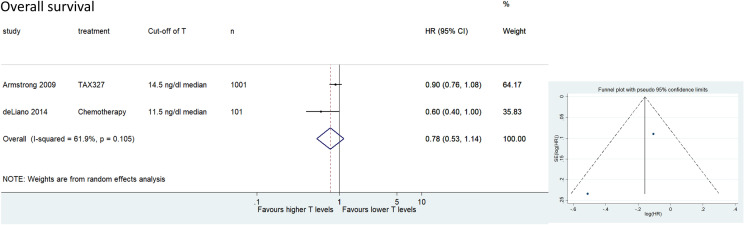
Forest and funnel plots showing the association of serum testosterone levels with overall survival in patients with castration-resistant prostate cancer treated with chemotherapy

Two cohorts including 1102 patients provided data on the association of testosterone with OS in patients with CRPC treated with chemotherapy. The forest plot (Fig. 4) revealed that higher testosterone levels were not significantly associated with better OS in patients with CRPC treated with chemotherapy (pooled HR 0.78, 95% CI 0.53–1.14; *z* = 1.29; *p* = 0197). The Cochrane’s *Q* test (*χ*^2^ = 2.62; *p* = 0.105) and *I*^2^ test (*I*^2^ = 61.9%) revealed significant heterogeneity. The funnel plot identified no cohort over the pseudo-95% CI (Fig. 4). Only one cohort provided data on the association of testosterone with PFS in patients with CRPC treated with chemotherapy [10]. This report showed that higher testosterone levels were significantly associated with worse PFS in patients with CRPC treated with chemotherapy (Table 1).

## Discussion

In this systematic review and meta-analysis, the prognostic effect of testosterone levels was assessed in patients with CRPC. Lower testosterone levels were found to be a poor prognostic factor for both PFS and OS in these patients. However, the results had some heterogeneity. One of the reasons could be the treatment differences. Therefore, performing subgroup analyses was necessary based on the treatment type for CRPC. Subgroup analyses based on the treatment type showed that higher testosterone was associated with better OS in CRPC patients treated with ARTAs, but not in those treated with chemotherapy.

Several studies have reported that a lower nadir serum testosterone level during primary ADT can be a favorable prognostic factor for hormone-sensitive PC [31, 32]. In contrast, in the CRPC disease state, we found that lower serum testosterone is significantly associated with worse PFS and OS. Claps et al suggested in their review that the relationship between serum testosterone level and PC prognosis varied in different clinical settings and according to ADT administration [12]. That is, these data suggest that patients who achieve the lowest testosterone levels during conventional first-line ADT generally benefit more from this treatment. However, after advancement to CRPC disease state, patients with lower testosterone levels seem to have a worse prognosis. Persistent blockage of androgen signaling is thought to trigger the selection of PCa cell clones that can upregulate the AR bypass pathway, ultimately conferring a castration-resistant phenotype [2]. Several studies have demonstrated AR bypass pathways are not dependent on AR for its downregulation [33]. It is speculated that low testosterone status may confer more aggressive biological and clinical conditions related to its survival and therapeutic response. More detailed studies are required in the future to understand this bipolar prognostic role of testosterone as patients experience disease progression from hormone-sensitive to castration-resistant disease states.

Theoretically, in patients with CRPC, persistent AR signaling partially deferred by relatively higher androgen level, are most likely to benefit from ARTAs. In fact, Efstathiou et al demonstrated that pretreatment tumor nuclear AR overexpression and CYP17 expression were associated with a worse sustained benefit of enzalutamide [34]. In a retrospective analysis of COU-AA-301, the median OS increased in a stepwise manner with increasing quartiles of pretreatment serum testosterone levels in patients with CRPC, irrespective of the administration of abiraterone acetate plus prednisone or prednisone alone [35]. Conversely, Shiota reported that AR signaling conferred therapeutic resistance to taxanes in vitro [36]. Furthermore, the author also found, based on clinical data, that higher pretreatment serum testosterone level in patients treated with taxane chemotherapy (ie. docetaxel and cabazitaxel) could predict a worse survival prognosis [10]. These findings and our meta-analysis suggest that serum testosterone levels, at the CRPC disease state, could not only be a prognostic factor for OS but also help identify which patients are likely to benefit from ARTAs versus taxane chemotherapies.

This study had several limitations. First, substantial heterogeneity was observed across the included studies. Therefore, we performed subsequent subgroup analyses to explore its potential causes. When we performed the stratification analyses in the ARTAs subgroup, heterogeneity was nonsignificant. Second, cut-off value for testosterone among the included studies was highly variable and not standardized. The most used cut-off value was the median value, but they varied among the included studies. Third, a possible confounding factor may have occurred as each study included different independent variables in the multivariable analysis. Fourth, in the included studies, only two mentioned the time when the blood samples were obtained. Since testosterone has diurnal variation, the measurement of serum testosterone is usually recommended between 7:00 and 11:00 a.m. maybe even in the late ADT cohorts [37]. Fifth, most of the data were retrospective, and there were many reports in which various treatment lines were mixed. Previous treatment may affect the response rate of next treatment. However, Sakamoto et al reported that TST levels remained as predictors even among the factors that included the previous usage of chemotherapy [9]. Finally, no patient in the study population had received upfront docetaxel or ARTAs as initial therapy for hormone-sensitive metastatic PCa. Therefore, the role of serum testosterone levels remains unclear in the patients receiving this new standard treatment. Interestingly, two studies reported that testosterone levels were useful in predicting the effects of ARTAs after docetaxel, so testosterone levels may be useful in cases following upfront docetaxel [28, 29]. Further investigations are required to clarify the role of testosterone as predictive biomarker in these patients [38].

This meta-analysis demonstrates that serum testosterone levels, at focus of CRPC, are predictive of oncologic outcomes and OS. In subgroup analyses, lower testosterone levels remained a poor prognostic factor for OS in CRPC patients treated with ARTAs, but not in those treated with chemotherapy. Therefore, testosterone levels could be a useful biomarker to identify a subgroup of patients who are likely to respond poorly to ARTAs in the CRPC setting. Validation through on diagnosis of data from well prospective trials could help establish solid evidence to include into guidelines and to fine true clinical decision making of ADT-alone treated patients who become CR.

## Electronic supplementary material

Below is the link to the electronic supplementary material.

Supplementary material 1 (TIF 58 kb)
