# Supplementary figures and images for: Prognostic value of testosterone for the castration-resistant prostate cancer patients: a systematic review and meta-analysis
Source: Int J Clin Oncol. 2020 Jul 17;25(11):1881–91. doi: 10.1007/s10147-020-01747-1 (PMC7572350; doi:10.1007/s10147-020-01747-1)

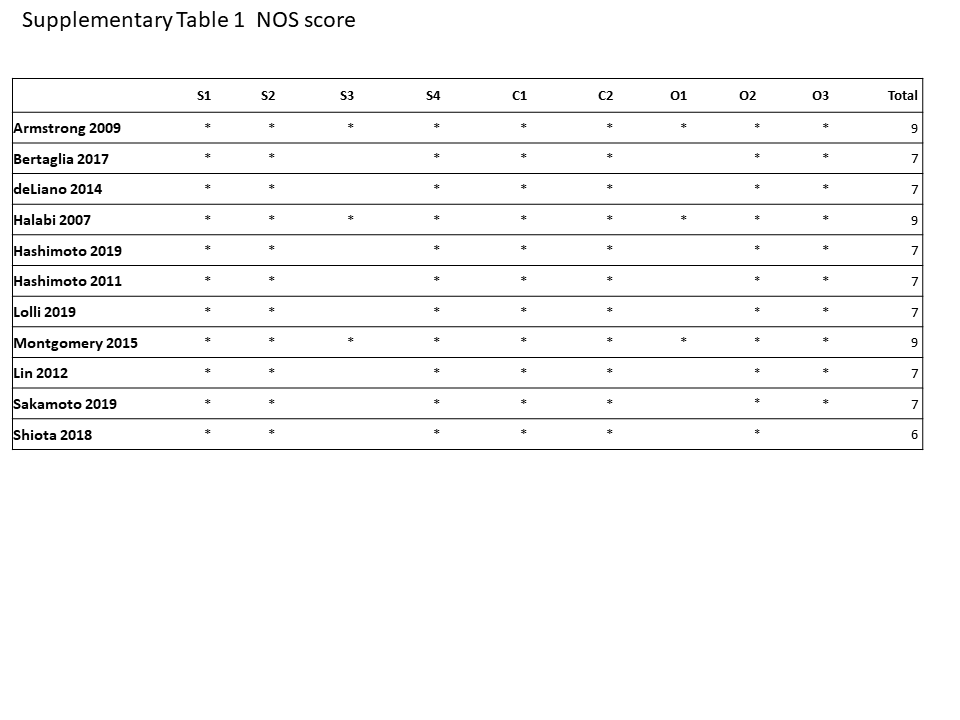

Supplement: Supplementary file 1 — Supplementary material 1 (TIF 58 kb) [file 10147_2020_1747_MOESM1_ESM.tif]
